# The clinical efficacy of first-generation carcinoembryonic antigen (CEACAM5)-specific CAR T cells is limited by poor persistence and transient pre-conditioning-dependent respiratory toxicity

**DOI:** 10.1007/s00262-017-2034-7

**Published:** 2017-06-28

**Authors:** Fiona C. Thistlethwaite, David E. Gilham, Ryan D. Guest, Dominic G. Rothwell, Manon Pillai, Deborah J. Burt, Andrea J. Byatte, Natalia Kirillova, Juan W. Valle, Surinder K. Sharma, Kerry A. Chester, Nigel B. Westwood, Sarah E. R. Halford, Stephen Nabarro, Susan Wan, Eric Austin, Robert E. Hawkins

**Affiliations:** 1 Department of Medical Oncology, The Christie NHS Foundation Trust, Manchester Academic Health Science Centre, Wilmslow Road, Withington, Manchester, M20 4BX UK; 2 Faculty of Biology, Medicine and Health, The University of Manchester, Manchester Academic Healthcare Science Centre, Wilmslow Road, Withington, Manchester, UK; 3 Clinical and Experimental Pharmacology Group, Cancer Research UK Manchester Institute, The University of Manchester, Wilmslow Road, Withington, Manchester, UK; 4 University College London (UCL) Cancer Institute, Paul O’Gorman Building, 72 Huntley Street, London, UK; 5 Cancer Research UK Centre for Drug Development, Angel Building, 407 St John Street, London, UK; 6 Stem Cells and Immunotherapy, National Health Service Blood and Transplant (NHSBT) Liverpool Centre, Speke, Liverpool, UK; 7 Present Address: Cellular Therapeutics Limited, Grafton Street, Manchester, UK

**Keywords:** CEA, Chimeric antigen receptor, T cells, Toxicity, Persistence

## Abstract

The primary aim of this clinical trial was to determine the feasibility of delivering first-generation CAR T cell therapy to patients with advanced, CEACAM5^+^ malignancy. Secondary aims were to assess clinical efficacy, immune effector function and optimal dose of CAR T cells. Three cohorts of patients received increasing doses of CEACAM5^+^-specific CAR T cells after fludarabine pre-conditioning plus systemic IL2 support post T cell infusion. Patients in cohort 4 received increased intensity pre-conditioning (cyclophosphamide and fludarabine), systemic IL2 support and CAR T cells. No objective clinical responses were observed. CAR T cell engraftment in patients within cohort 4 was significantly higher. However, engraftment was short-lived with a rapid decline of systemic CAR T cells within 14 days. Patients in cohort 4 had transient, acute respiratory toxicity which, in combination with lack of prolonged CAR T cell persistence, resulted in the premature closure of the trial. Elevated levels of systemic IFNγ and IL-6 implied that the CEACAM5-specific T cells had undergone immune activation in vivo but only in patients receiving high-intensity pre-conditioning. Expression of CEACAM5 on lung epithelium may have resulted in this transient toxicity. Raised levels of serum cytokines including IL-6 in these patients implicate cytokine release as one of several potential factors exacerbating the observed respiratory toxicity. Whilst improved CAR designs and T cell production methods could improve the systemic persistence and activity, methods to control CAR T ‘on-target, off-tissue’ toxicity are required to enable a clinical impact of this approach in solid malignancies.

**Electronic supplementary material:**

The online version of this article (doi:10.1007/s00262-017-2034-7) contains supplementary material, which is available to authorized users.

## Introduction

CAR T cell technology has risen in prominence as a result of the durable, objective clinical responses reported in early phase trials testing CD19 CAR T cells against B cell leukaemia [1–6]. CAR’s typically consist of scFv tumour targeting domains fused to T cell signalling receptors that, when expressed in a T cell, can effectively re-direct immune effector activity towards the cell surface target antigen specified by the scFv domain and independent of HLA restriction [7–10]. Initial testing of CAR T cell therapy against solid tumours has proven to be less efficacious [11–14]. These early clinical trials employed first-generation CAR technology and no patient pre-conditioning. Against this background, we conceived a trial design that questioned the role of CAR T cell dose and the relative intensity of patient pre-conditioning upon the function and clinical impact of adoptively transferred CAR T cells.

The tumour-associated antigen explored in this trial was carcinoembryonic antigen (CEA; CEACAM5; CD66e) which is expressed at high levels in a broad range of tumours including those of the gastrointestinal tract and has been extensively explored as a cancer vaccine target [15]. A phage-selected CEACAM5-specific scFv (MFE23), shown to be well tolerated in imaging and antibody-directed pro-drug therapy strategies [16–18], was fused to CD3ζ to generate a first-generation CAR termed MFEζ that was extensively characterized for structure and function in T cell lines and primary human T cells [19–23]. The in vivo anti-tumour activity of anti-CEACAM5 CAR T cells [24] further supported a strategy of targeting CEACAM5 within the context of a clinical trial.

The trial proposal involved dose-escalation of CAR T cells within 3 cohorts to reach a maximum dose of 5 × 10^10^ total T cells with fludarabine pre-conditioning and systemic IL2 support. Subsequent cohorts were to receive the maximum dose of MFEζ CAR T cells combined with an increased intensity of pre-conditioning delivered by the combination of cyclophosphamide and fludarabine [25]. The trial opened in 2007 after significant delays during the regulatory process due to issues reported in other immune-based trials [26, 27]. Fourteen patients were recruited prior to early termination due to transient acute toxicity after completion of cohort 4. We now report the clinical and scientific observations that confirm that intensity of pre-conditioning impacts upon the relative frequency but not absolute number of systemic MFEζ CAR T cells. Furthermore, systemic cytokine data imply immune activation of first-generation MFEζ CAR T cells in vivo, whilst evidence of raised IL-6, paralleling that seen in CD19 CAR T cell trials targeting B cell leukaemia [5], putatively implies a common mechanism of in vivo CAR T activity that is dependent upon patient pre-conditioning and, potentially, all generations of CAR design.

## Materials and methods

### Trial design

This was a single-centre open-label, dose-escalation Phase I study (ClinicalTrials.gov identifier NCT01212887) managed and conducted in accordance with the principles of Good Clinical Practice and UK legislative requirements (Medicines and Healthcare Regulatory Agency).

Primary objectives were to evaluate the feasibility of MFEζ CAR T cell therapy in patients with CEACAM5^+^ tumours, to assess toxicity and to determine dose of MFEζ CAR T cells for optimal survival in the circulation. Secondary objectives were to assess functionality of MFEζ CAR T cells isolated from the circulation, to obtain preliminary evidence of radiological response and to evaluate safety.

The trial was based on a 3 + 3 design for cohorts 1 to 3 and 4 + 3 for cohorts 4 and 5 (Table 2). If a dose-limiting toxicity was experienced, the cohort was to be expanded to six patients. All patients received pre-conditioning chemotherapy, MFEζ T cells then intravenous IL2 therapy. Patients in cohorts 1–3 received fludarabine chemotherapy (25 mg/m^2^/day for 5 days) with inter-cohort escalation of MFEζ T cells. Patients in cohort 4 received maximum MFEζ T cell dose with cyclophosphamide (60 mg/kg/day for 2 days) prior to fludarabine (25 mg/m^2^/day for 5 days) chemotherapy. All patients received IV IL2 (600,000 IU/Kg 15-min infusion every 8 h maximum 12 doses). IL2 was commenced 90 min after MFEζ T cells. Criteria for IL2 dose delay, reduction or discontinuation defined within the protocol resulted in administration of a variable number of IL2 doses.

Inclusion criteria for this study included patients with advanced, histologically confirmed CEACAM5^+^ malignancy where standard curative or palliative measures were not applicable, ≥18 years old, life expectancy over 3 months, performance status of 0 or 1, adequate renal, cardiac, haematological and biochemical function. Exclusion criteria included anti-cancer systemic treatment or radiotherapy within four weeks, on-going significant toxicity from previous therapies, brain metastases, significant non-malignant disease (including autoimmune disease), prior BMT, previous extensive radiotherapy, current other malignancies and patients taking, or likely to require systemic steroids or other immunosuppressants.

Adverse event (AE) monitoring commenced from the point of written consent. AEs were reported as per Common Terminology Criteria for Adverse Events (CTCAE) Version 3.0. The following dose-limiting toxicities were defined when they were almost certainly or probably drug related; toxicity ≥grade 3 as a result of MFEζ T cells; toxicity caused by MFEζ T cells or chemotherapy preventing commencement of IL2 within 24 h; toxicity ≥3 during IL2 therapy that did not resolve to ≤grade 2 within 48 h of stopping IL2; toxicity ≥grade 3 as a result of chemotherapy despite optimal supportive medication excluding bone marrow suppression.

Patients were treated as inpatients and discharged home when clinically appropriate. They were followed up as outpatients and underwent computerized tomography (CT) scans at 6 weeks, 3, 6 and 12 months which were reported to RECIST version 1.0.

### Production of MFEζ CAR T cells

MFEζ CAR T cells were produced in compliance with Good Manufacturing Practice as previously described [28].

### Blood collection, processing and cell counts

Blood samples were collected at pre-treatment, day 0 pre-infusion, 2, 6 h, days 1, 2, 3, 4 and 5 post infusion and weeks 1, 2, 3, 4, 5, 6, 12 and then 12 weekly until off trial. Within 24 h of blood draw, plasma and PBMCs were isolated from an EDTA blood at each time point following standard procedures and stored at −80 °C and in liquid nitrogen. An additional CPT™ Vacutainer tube [Becton–Dickinson (BD), NJ, USA] was collected at each time point for mononuclear cells isolation and gDNA extraction using a Wizard^®^ Genomic DNA Purification Kit (Promega, WI, USA) following the manufacture’s protocol. Blood counts were collected daily during hospitalization and at each visit using a certified clinical haematology service. All sample processing and subsequent assays were performed in compliance with good clinical laboratory practice guidelines and subjected to independent quality assurance control.

### Laboratory assays

#### Real-time PCR quantification of transduced cells

A validated quantitative PCR assay (qPCR) was developed to quantify the level of MFEζ CAR T cells in patient samples. A CAR-specific qPCR amplicon (MFEζ F primer 5′-CTTATTACTGCCAGCAAAGGAGTAGTT, R primer 5′-CAAAGCTCGCTCCGTCTGTAG, probe FAM-5′-CCCACTCACGTTCGGTGCTGGC) and genomic standard qPCR amplicon (b2 M, F primer 5′-GGAATTGATTTGGGAGAGCATC, R primer 5′-CAGGTCCTGGCTCTACAATTTACTAA, probe FAM-5′-AGTGTGACTGGGCAGATCATCCACCTTC) were used to determine total genome copies (b2 M) and transduced genome copies (MFEζ) per sample.

The assay was validated using a standard curve generated from a single-cell-cloned Jurkat-MFEζ cell line (100%) diluted to 10, 1, and 0.1% with non-transduced Jurkat gDNA. Each assay included a positive control of known transduction level (4%) and a non-transduced (0%) negative control. The acceptance criteria for the qPCR assay were set as Standard Curve *R* ^2^ value ≥0.95, positive control = 4% (±2%) and lower limit of detection = 0.1% transduced cells.

#### IFNγ ELISA analysis

A 96-well ELISA plate was coated for 2 h at 37 °C or overnight at 4 °C with 1 µg/ml IFNγ capture antibody (MAB-285, R&D systems, MN, USA) and then washed with PBS + 0.05% Tween. IFNγ standards were then added (200–0.5 pg/ml) along with 10 and 100 µl patient plasma for each sample. Following incubation at 37 °C for 1 h, 100 µl of biotinylated IFNγ detection antibody (BAF-285, R&D systems, MN, USA) was added to each well for a further hour at 37 °C. After three washes, Streptavidin peroxidase (POD) conjugate (Roche, Basel, Switzerland) was then added and the plate incubated at 37 °C for 30 min and then POD blue substrate (Roche, Basel, Switzerland) added for 30 min. The reaction was stopped by the addition of H_2_SO_4_ and the plate read at 450 nm. The concentration of IFNγ was calculated using the standard curve.

#### Determination of cytokine concentrations in serum samples by Luminex bead array

Concentrations of plasma cytokines were measured using the Bio-Plex Pro^™^ Human Cytokine 17-plex Assay kit (Bio-Rad Laboratories Inc, California, USA). Reconstituted standards, cytokine-specific coupled beads, detection antibodies and 50 µl of thawed serum samples were combined according to manufacturer’s instructions and data acquired with the Bio-Plex^™^ 200 reader (Bio-Rad Laboratories Inc, California, USA). Data were analysed using Bio-Plex Manager™ software v6.0 (Bio-Rad Laboratories Inc, California, USA).

#### Anti-mouse scFv assay

96-well microtiter plates were coated with MFE antibody (1 µg/ml in carbonate-bicarbonate buffer, 100 µl/well) for one hour at room temperature, washed with PBS and blocked with 5% Marvel/PBS/Tween solution (150 µl per well). After washing, the wells with incubated with either positive, negative control, PBS or patient samples diluted in 1% Marvel/PBS/Tween solution (1/100 dilution, 100 µl/well) in four replicates for 1 h. The wells were washed and incubated with 100 µl per well of the appropriate secondary antibodies diluted in 1% Marvel/PBS/Tween solution. For the patient samples, rabbit anti-human IgG was added for 1 h. The wells were washed and incubated with anti-rabbit HRP antibody (100 µl/well for 1 h). After washing, 100 µl/well substrate (o-phenylenediamine in phosphate citrate buffer) was added and the reaction stopped after 5 min by adding 4 M hydrochloric acid (50 µl/well). The OD of the wells was obtained at 490 nm. The results were recorded as positive or negative according to previously set criteria for human anti-MFE antibody assay.

### CEACAM5 expression in the lung

Following discontinuation of the clinical trial, we assessed whether the observed respiratory symptoms could have been attributable to CEACAM5 expression in the lungs of the patients. We accessed nine lung tissue samples from Manchester Cancer Research Centre Biobank. Eight were non-cancerous tissue from patients undergoing resection for lung cancer and the ninth was from a patient with metastatic colorectal cancer undergoing a lung resection. IHC using the Col-1 antibody [29] and qPCR were used to explore CEACAM5^+^ expression in these samples.

## Results

### MFEζ trial background and recruitment

Of the twenty-three patients who gave informed consent, eight failed screening and one patient was ineligible due to disease progression prior to therapy. The remaining 14 patients had a range of metastatic gastrointestinal malignancies (Table 1). Each patient received pre-conditioning chemotherapy, MFEζ T cells and systemic IL2 support in 4 cohorts (Table 2). The dose of MFEζ T cells received by patient 36007 in cohort 2 was lower than stipulated for full evaluation within the cohort so an additional patient (36009) was recruited as a replacement.

**Table 1:** Patient demographics

Cohort	Patient no (sequentially treated)	Sex	Age	Primary diagnosis (adenocarcinoma unless otherwise stated)	Metastatic sites	WHO PS	Previous treatment
1	36003	M	66	Colon	Bone, liver, lung	1	S, RT right hip Ir5FU, CapOx, Ox5FU, Ir5FU, Palliative RT R Hip
	36004	M	47	Stomach	Liver, lymph node	1	ECX, ECX rechallenge
	36005	M	63	Rectum	Liver, lung	1	S, CapOx, IrCap, Ir Cetuximab
2	36006	F	36	Pseudomyxoma peritonei	Spleen	1	S, Mitomycin + Capecitabine 3 challenges
	36007	M	53	Stomach	Liver, lymph node	1	ECX
	36008	F	49	Stomach	Chest wall, liver, lymph node, peritoneum	0	S, ECX
	36009	M	41	Rectum	Bone, liver, lung	0	Ox5FU + Bevacizumab, 5FU + Bevacizumab, Ir5FU + Bevacizumab, Ox5FU + Bevacizumab
3	36010	M	41	Oesophagus	Lymph node	1	ECX
	36011	M	49	Oesophagus	Lymph node		EOX
	36012	F	42	Gastro-oesophageal junction	Liver, lung, lymph node	1	EOX
4	36013	F	40	Pancreas	Liver, lung, peritoneum	1	S, RT local recurrence, GemCap, Capecitabine
	36014	F	45	Colon	Liver	0	S, metastasectomy (lung and liver), CapOx, Ir5FU, Ox5FU, IrCap.
	36015	F	56	Colon	Muscle, ovary, peritoneum,	0	S, Ox5FU
	36017	M	40	Caecum	Liver, lung	1	S, CapOx, Irinotecan

*S* surgical resection of primary disease, *RT* palliative radiotherapy, *Ir5FU* irinotecan plus 5Fluorouracil, *CapOx* capecitabine plus oxaliplatin, *Ox5FU* oxaliplatin plus 5Fluorouracil, *ECX* epirubicin, cisplatin plus capecitabine, *IrCap* irinotecan plus capecitabine, *EOX* epirubicin, oxaliplatin plus capecitabine

**Table 2:** Cohort treatment outline, CAR T cell dose and patient outcome

Cohort	Patient number	Chemotherapy administered	No of IL2 infusions^a^	Cohort dose (total T cells per patient)	Actual Dose of viable T cell product (×10^10^)	MFEζ Transgene expression (%)	Total dose of viable MFEζ T cells administered (×10^9^)	Best tumour response^b^
1	36003	F	7	10^9^	0.10	21.3	0.21	SD (150)
	36004	F	12		0.10	21.4	0.21	SD (108)
	36005	F	11		0.10	22.1	0.22	PD
2	36006	F	5	10^10^	0.97	27.3	2.64	SD (83)
	36007	F	3		0.41	33.7	1.38	PD
	36008	F	3		1.00	26.4	2.64	PD
	36009	F	8		1.00	24.5	2.45	PD^c^
3	36010	F	3	10^9^ – 5 × 10^10^	1.01	20.1	2.03	SD (88)
	36011	F	2		1.21	23.6	2.86	PD
	36012	F	5		0.98	34.9	3.43	PD
4	36013	F + C	7	10^9^ – 5 × 10^10^	0.90	37.3	3.36	SD (84)
	36014	F + C	3		1.32	29.4	3.89	SD (155)
	36015	F + C	5		0.68	36.4	2.48	SD (119)
	36017	F + C	10		0.17	20.1	0.33	PD

*F* fludarabine 25 mg/m^2^/day for 5 days, *C* cyclophosphamide 60 mg/kg/day for 2 days

^a^Intravenous bolus IL-2; 600,000 IU/kg per dose

^b^The duration of stable disease (SD) has been estimated from the pre-treatment scan date to the most recent scan on study. Progressive disease as assessed at week 6 scan

^c^Clinical progression (patient unfit for CT restaging scan)

### Clinical efficacy

No patients attained an objective response by RECIST although several had marked reduction in tumour markers. Seven patients achieved stable disease as their best response 6 weeks post MFEζ T cell infusion with 3 patients maintaining this response at 12 weeks (Table 2). There were no long-term sustained responses and thirteen patients died of progressive disease. One patient (36014) remained alive 56 months post infusion. This patient achieved disease stabilization as best response but progressed 155 days after baseline scan (127 days post T cell infusion) and went on to receive other therapy.

### Increased intensity of pre-conditioning chemotherapy improves the systemic engraftment but not absolute number of MFEζ CAR T cells

**Fig. 1:**
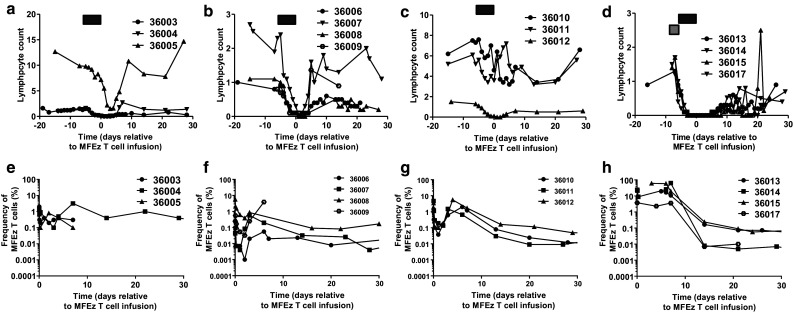
Increased intensity of patient pre-conditioning results in prolonged lymphodepletion and increased frequency of MFEζ CAR T cell engraftment. Lymphocyte counts (×10^9^ cells/L) of patients in **a** cohort 1, **b** cohort 2, **c** cohort 3 and **d** cohort 4. The timing of fludarabine pre-conditioning (*black box*) and cyclophosphamide (*grey box*) are shown prior to MFEζ T cell infusion on day 0. MFEζ CAR T cell frequency determined by qPCR in blood samples estimated by comparison to β2-microglobulin to determine relative CAR T cell frequency in **e** cohort 1, **f** cohort 2, **g** cohort 3 and **h** cohort 4

Patients in cohorts 1–3 experienced a transient decrease in whole blood counts (Supplementary Fig. 1a–c) lasting approximately one week with a period of lymphodepletion lasting 2–3 days with a nadir generally 1–2 days post cell infusion (Fig. 1a–c). Similar kinetics in monocyte and neutrophil counts were observed (Supplementary Fig. 2a–f). By contrast, patients in cohort 4 experienced a prolonged period of depressed blood counts (Supplementary Fig. 1d) and lymphopenia lasting approximately 10 days beginning 2 days prior to cell infusion (Fig. 1d). Lymphocyte counts started to return towards normal levels from 10 days after cell infusion, a trend mirrored in the monocyte and neutrophil compartments although a rebound increase in both cell types was briefly observed during peripheral reconstitution (Supplementary Fig. 2g–h).

Molecular analysis of peripheral blood samples confirmed MFEζ engraftment levels in the region of 0.1–5% in cohorts 1–3 with a general trend of higher frequency of transduced cells correlating with infused cell dose across the cohorts (Fig. 1e–g). Engraftment levels peaked around day 6 before dropping to undetectable levels in 2 out of 3 patients in cohort 1. After a peak of similar magnitude to cohort 1, only very low engraftment levels (in the region of 0.01%) were maintained in the majority of patients in cohorts 2 and 3 up to day 30. By contrast, patients in cohort 4, despite receiving a wide range of T cell doses (range 1.7 × 10^9^–1.3 × 10^10^ total T cells, 20–37% transduced), all achieved much higher levels of MFEζ T cell engraftment at time points immediately after infusion reaching up to 60% within 5–10 days post infusion (Fig. 1h). However, as with earlier cohorts, a rapid drop off with no significant persistence of MFEζ T cells observed two weeks after T cell transfer. Despite the percentage difference in CAR T cell engraftment, when the number of MFEζ T cell numbers were estimated by multiplying the fraction of transgene-positive T cells from the qPCR data and the absolute lymphocyte counts, there was no significant difference in the absolute number of CAR cells between cohorts 3 and 4. This implies dissociation between intensity of pre-conditioning and the absolute number of systemic CAR T cells albeit clearly within a limited number of patients within the two cohorts (Supplementary Fig. 3a–b).

### Serum tumour markers and cytokine levels

**Fig. 2:**
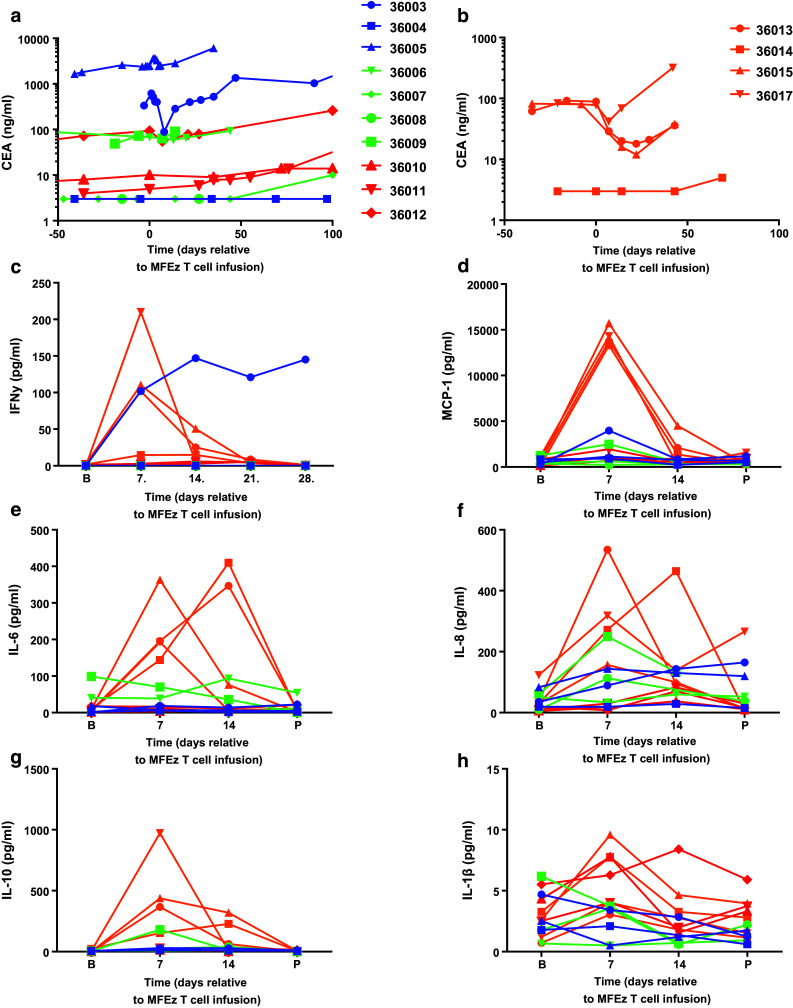
Consistent decrease in systemic CEACAM5 levels and transient elevations in serum cytokines are seen in cohort 4 post-MFEζ CAR T cell infusion. Patients in each cohort are colour-coded to identify cohorts: cohort 1 in *blue*, cohort 2 in *green*, cohort 3 in *red* and cohort 4 in *orange*. Serum CEACAM5 levels were determined in patient blood samples prior and post-MFEζ CAR T cell infusion for **a** cohorts 1–3 and **b** cohort 4. The same colour-coding is applied to serum cytokine analysis of **c** IFNγ where analysis was performed at baseline (**b**) and weekly until day 28. For all other cytokines, **d** MCP-1, **e** IL-6, **f** IL-8, **g** IL-10 and **h** IL-1β where baseline, day 7, day 14 and post-treatment (P; day 21 or 28) are shown

Within cohorts 1–3, no significant reduction in serum CEA was seen at any stage during the trial except for a transient drop between MFEζ T cell infusion and week 4 for 1 patient (36003; Fig. 2a). However, the three patients in cohort 4 who had significant baseline levels of soluble CEA (36013, 36015, 36017) were all found to have a reduction in levels post-infusion (Fig. 2b). In two patients (36013, 36015), this level remained below that seen pre-infusion for at least 6 weeks. Six patients across all cohorts demonstrated a transient reduction in CA19-9 levels post-infusion (Supplementary Fig. 4); however, responses were again short-lived. There was no obvious correlation between a biochemical response to therapy and clinical outcome.

In terms of systemic cytokine levels, patients within cohort 4 displayed peak levels of IFNγ, monocyte chemotactic protein-1 (MCP-1), IL-6, IL-8 and IL-10 on day 7–14 correlating with peak frequency of MFEζ CAR T cell engraftment that returned to baseline at progression but with no effective modulation of the levels of IL-1β (Fig. 2c–h). Interestingly, the first patient treated (36003) with the lowest dose of T cells and fludarabine pre-conditioning had a sustained systemic level of IFNγ (Fig. 2c), a suggestion of raised levels on day 7 of MCP-1 post-MFEζ and an apparent transient drop in serum CEA (Fig. 2a) with no other obvious elevation of other cytokines monitored.

### Anti-mouse scFv humoral immune response

Since anti-CAR transgene immune responses were implicated in a previous CAR T cell trial [13], human anti-mouse antibody (HAMA) responses were assessed. No HAMA response was seen in any patient above that of the pre-established lower limit of detection (Supplementary Fig. 5).

### Preliminary evidence of MFEζ T cell trafficking into tumour sites

**Fig. 3:**
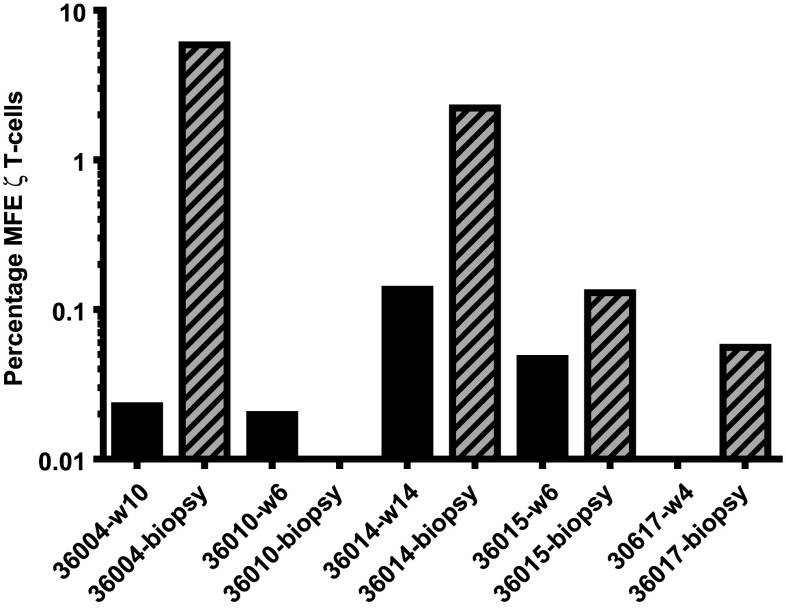
Molecular analysis of fine needle biopsy samples to determine the presence of MFEζ CAR T cells within tumour tissue. MFEζ transgene in tumour biopsies (*hatched bars*) and peripheral blood (*black bars*) taken at the same time as the biopsy at week 10 for patent 36004; week 6 for patient 36010; week 14 for patient 36014; week 6 for patient 36015; and week 4 for patient 36017

Fine needle biopsies taken from a restricted number of patients (36004, 36010, 26014 and 36017) were analysed for the presence of MFEζ T cells by qPCR with the frequency of CAR T cells determined by comparison to the β2-microglobulin control. A strong MFEζ transgene signal detected in a tumour biopsy collected from patient 36,004 was at much higher level than that detected in peripheral blood at an equivalent time point (Fig. 3). The MFEζ transgene signal was also detected in biopsy samples from patients 36014, 36015 and 36017 at higher levels than blood samples taken at the same time as the biopsies (Fig. 3). However, no specific signal was detected a biopsy taken from patient 36010. The limiting quantity of biopsy material restricted further analysis; however, these data suggest that MFEζ T cells possess some capacity to migrate into sites of tumour.

### Adverse events

**Fig. 4:**
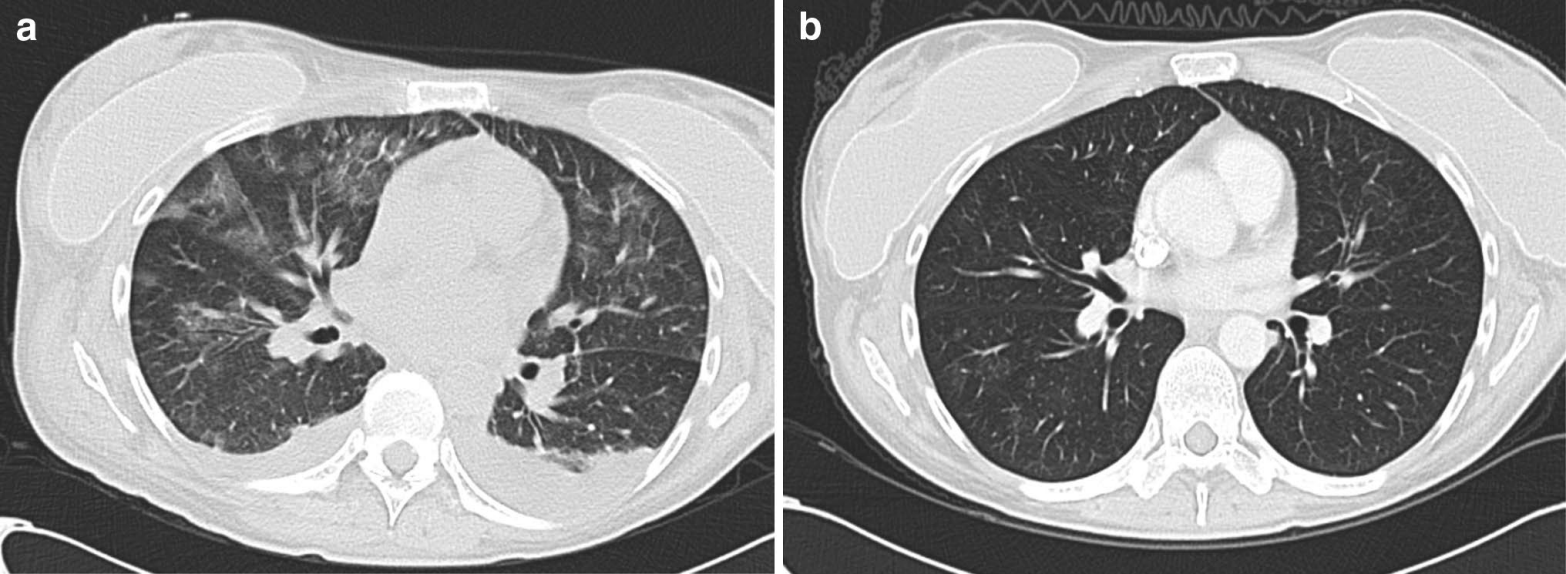
Evidence of pulmonary infiltrates consistent with local cytokine release syndrome in patient 36,013. CT images demonstrating non-specific pulmonary infiltrates 10 days after MFEζ T cell infusion (**a**) and resolution by day 42 (**b**)

All patients experienced AEs with grade ≤2 events resolving within ten days. Patients in cohorts 1–3 experienced grade ≥3 level toxicities consistent with fludarabine and IL2 therapy (Supplementary Table 1). Patients in cohort 4 experienced similar toxicities albeit with more pronounced grade and duration as anticipated for the level of pre-conditioning chemotherapy. Each patient in this cohort also experienced additional AEs after cell transfer not readily attributable to chemotherapy or IL2 (Supplementary Table 2). The first patient treated in cohort 4 (36013) developed grade 3 tachypnea and pulmonary infiltrates which resolved within 18 days (Fig. 4). Patient 36014 developed respiratory distress 5 days following T cell infusion and admitted to the critical care unit. All symptoms resolved as confirmed by a repeat CT scan one month later. Similar but less dramatic pulmonary toxicities were seen both of the subsequent patients in cohort 4 (36015 and 36017). The respiratory toxicity was managed conservatively with supportive measures including high-flow oxygen. No patients required invasive ventilation and, whilst prophylactic antibiotics were given, there was no confirmation of respiratory infection and systemic steroids were not required. These events were all reported as a suspected unexpected serious adverse reaction (SUSAR). Three other SUSARs were also reported during the study including grade 3 neutropenic sepsis (36013), grade 3 decreased appetite (36006) and grade 4 neutropenic sepsis and grade 2 intracranial haemorrhage in patient 36,008. AEs completely resolved within 18 days.

At the completion of cohort 4, a safety review conducted by the trial sponsor concluded that the respiratory toxicities observed across all patients in cohort 4 posed an unfavourable risk benefit profile to patients. This toxicity combined with the lack of demonstrable clinical efficacy and the lack of sustained CAR T cell engraftment in cohorts 1–4 suggested to the sponsor that achieving the primary endpoint of prolonged MFEζ T cell engraftment was unlikely to be met in subsequent cohorts. This resulted in the early closure of the study.

### CEACAM5 expression in the lung

IHC identified two samples (W0076T1PNf and W0096T1PNc) as strongly positive with frequent CEACAM5^+^ cells present and three intermediately positive (W00115T1PNd, W0098T1PNf and W00851PNc) with less frequent immuno-positive cells present which was confirmed by qPCR analysis (Supplementary Fig. 6). In the remaining three samples, there was either weak antibody staining or weak qPCR signal questioning the level of CEACAM5 expression in these samples (Supplementary Fig. 6). Thus, there appeared to be strong/intermediate CEACAM5 expression in 5/8 non-cancer lung resection samples. Interestingly, in one further lung resection sample (W0094T1PNc) from a patient with metastatic colorectal cancer, there was no qPCR signal at all for CEACAM5 and very low-level staining by the anti-collagen 1(Col-1) antibody (Supplementary Fig. 6).

## Discussion

The primary end point of assessing the feasibility of delivering MFEζ CAR T cell therapy was achieved with all fourteen eligible patients receiving pre-conditioning, CAR T cells and at least two doses of IL2. However, there were challenges in meeting the higher T cell dose required in cohorts 3 and 4 where the seven patients received 0.9 ± 0.4 × 10^10^ T cells that was below the proposed maximum T cell dose of 5 × 10^10^ T cells. The GMP compliant production methods used in this trial were established over ten years ago [28] and current production methods including bioreactor technology [30] now enable the routine production of high T cell numbers in manageable culture volumes.

Whilst the trial failed to identify a CAR T cell dose that resulted in long-term persistence of the MFEζ T cells, it did confirm that an increased intensity of pre-conditioning enhanced the relative frequency of CAR T cell engraftment. Furthermore, the intensity of chemotherapy was also critical for CAR T cell function as cytokines pertinent to T cell activation were only consistently detected in cohort 4. However, there was no obvious difference in the absolute numbers of systemic MFEζ T cells suggesting a lack of in vivo CAR T cell expansion that is likely to adversely impact upon the therapeutic power of the approach.

Aside from sub-optimal culture technology, current evidence suggests that second and subsequent generations of CARs can deliver increased potency of T cell signalling, persistence and anti-tumour activity [10, 31, 32]. A recent report of hepatic artery infused second-generation CEACAM5-specific CAR T cells with systemic IL2 and no patient pre-conditioning reported tissue localization of the CAR T cells but no evidence of prolonged persistence and limited evidence of CAR T cell effector function albeit within the early stages of the overall clinical trial [33]. Undoubtedly, CD19 CAR T cells benefit from the ready access to antigen^+^ leukaemic target cells resident within the periphery that can engage the CAR and help to drive T cell persistence. CAR T cells targeting solid tumour antigens clearly require greater help which currently includes intensive pre-conditioning but will also require additional strategies to enhance localization and challenge the strongly immune-suppressive tumour microenvironment. More recent engineering strategies such as the armoured CAR approach [34] to alter the balance of immunity within the tumour potentially enhance clinical response.

A critical issue in this trial was the transient acute respiratory toxicity observed in patients within cohort 4 which combined with the lack of prolonged high levels of CAR T cell persistence and an absence of clinical response resulted in the early termination of the trial. Our expectation before opening of the trial was the potential for bowel toxicity due to the expression of CEACAM5 within the intestine [35] though no evidence of bowel-related toxicity was seen. We hypothesized that the respiratory symptoms observed in patients in cohort 4 may be indicative of ‘on-target off-tumour’ MFEζ T cell binding to CEACAM5 antigen present within the lung which would be consistent with the affinity (2.5 nM) of the MFE23 scFV [36] augmented by avidity in CAR T format. There are contradictory reports in the literature with respect to CEACAM5 expression in normal lung with some documenting relatively high levels of CEACAM5 expression in normal lung tissue [37]; however, cross-reactivity of CEACAM5-specific monoclonal antibodies has raised questions whether the detected proteins are CEACAM5 or related CEA-family members [29]. Our assessments demonstrated CEACAM5 expression in 5 of 9 non-cancer lung resection samples supporting the hypothesis that MFEζ T cell binding to CEACAM5 antigen present within normal lung may have contributed to this toxicity (Supplementary Fig. 6). A recent study investigating non-cancer tissue sections taken from patients with lung cancer identified the clear expression of CEACAM5 as well as CEACAM1 and CEACAM6 family members in this non-cancer tissue [38]. This report also demonstrated that IFNγ up-regulates CEACAM5 expression on normal bronchiolar epithelial cells. Thus, CAR T cell activation may drive a local feedback loop through cytokine production up-regulating CEACAM5 expression and driving local toxicity. This is consistent with the timing of the toxicity seen where no symptoms were manifest shortly after T cell infusion which would have been consistent with acute reaction with pre-existing low levels of antigen as was seen in the acute death reported with a Her2-targeted CAR trial [39]. The delayed toxicity (peak around day 5–7 post infusion) coinciding with peak of transduced T cells in the blood and the peak IFNγ is consistent with cytokine release and/or with delayed lung recognition because of increased MFEζ CAR T cells and/or up-regulation of CEACAM5 as a result of exposure to IFNγ [38]. The delayed nature of the respiratory toxicity would also steer away from the likelihood of it being attributable solely to the IL2 and/or the pre-conditioning chemotherapy, although the possibility that one or both of these contributed cannot be excluded.

The transient nature of the toxicity may reflect the poor persistence of the MFEζ CAR T cells. Importantly, all cohort 4 patients were managed conservatively with supportive measures only and no immune modulation such as steroids and yet fully recovered. Although the toxicity was transient, immune modulation may have reduced the severity of toxicity but also likely to reduce engraftment and efficacy, thereby impacting upon the viability of the therapy.

Aside from IFNγ, cohort 4 patients also had elevated levels of IL-6, IL-8 and MCP-1 coupled with little modulation of IL-1β that reflects observations made in B-ALL patients receiving CD19 CAR T cells [5]. This suggests that cytokine release including IL-6 can occur with the most basic CAR design and is dependent upon intensive pre-conditioning regimes since no respiratory-related symptoms or elevated cytokine levels were seen in cohort 3 patients. In further experiments, we attempted to recapitulate this transient toxicity through the infusion of CEA transgenic mice with high doses of MFEζ CAR T cells combined with high dose IL2 (data not shown). There was no evidence of adverse toxicities observed in these animals illustrating the limitations of pre-clinical models to accurately model the human in vivo situation.

The lack of bowel toxicity in this trial contrasts with the severe colitis seen with T cells armed with TCR specific for CEACAM5 [40]. Presumably, this reflects the marked polarized expression of cell surface CEACAM5 that is only seen in the luminal surface of the bowel [35], thus making it relatively immune-protected from T cell cytotoxic activity as compared to the non-polarized expression profile of peptide-bound HLA molecules. The pulmonary toxicity in our trial serves to illustrate the challenges in isolating specific causes of toxicity in adoptive cell therapy where many will be multifactorial and inter-related and where there are multiple variables across different trials. These variables range from the pre-conditioning chemotherapy, IL2 regime (if used), variation in the scFv affinity for its target, dose of cells, manufacturing differences, to name but a few.

No significant treatment-related neurological toxicity was noted in this trial where all patients received a moderate dose of fludarabine (25 mg/m^2^/day for 5 days) with first-generation CAR T cell therapy. However, there have been a number of reports of significant neurologic toxicities observed in CAR T cell trials. Notably a recent trial sponsored by Juno Therapeutics was halted after a number of patients died having developed cerebral oedema attributed initially to intensified lymphodepleting chemotherapy with fludarabine [41]. The trial was allowed to resume without the intensified chemotherapy, but this was not sufficient to ameliorate the toxicity as further patient deaths subsequently occurred. Whilst the significant toxicity observed in the Juno trial is not mirrored in our trial, the details of the mechanism of the toxicity will have an important influence on the direction for the field as a whole.

To conclude, this trial underlines the importance of pre-conditioning chemotherapy for CAR T cell therapy and highlights the need to design CAR T cells to maximize the discrimination between high-level target expression within the tumour and low level within some normal tissues to avoid toxicity. The powerful avidity effect of CAR T cells means that lower affinity antibodies for CAR T cell therapy may provide better discrimination between low-level expression on normal tissue and high level on the tumour. However, where such discrimination is not possible and clinical benefits are seen, approaches such as local steroids to manage on-target, off-tumour toxicity will be essential to fully explore the therapeutic potential of this approach in patients with solid tumours. CEACAM5 remains a potentially useful target for CAR T cells with those caveats.

### Electronic supplementary material

Below is the link to the electronic supplementary material.

Supplementary material 1 (PDF 815 kb)

## Abbreviations

AE: Adverse event
CAR: Chimeric antigen receptor
CEA: Carcinoembryonic antigen (also CEACAM5; CD66e)
Col-1: Anti-collagen 1 antibody
CT: Computerized tomography
CTCAE: Common toxicity criteria for adverse events (version 3.0)
HAMA: Human anti-mouse antibody
MCP-1: Monocyte chemotactic protein-1
POD: Peroxidase
qPCR: quantitative polymerase chain reaction
SUSAR: Suspected unexpected serious adverse reaction
