# Supplementary material for: The clinical efficacy of first-generation carcinoembryonic antigen (CEACAM5)-specific CAR T cells is limited by poor persistence and transient pre-conditioning-dependent respiratory toxicity
Source: Cancer Immunol Immunother. 2017 Jun 28;66(11):1425–36. doi: 10.1007/s00262-017-2034-7 (PMC5645435; doi:10.1007/s00262-017-2034-7)
Supplement: Supplementary file 1 — Supplementary material 1 (PDF 815 kb) [file 262_2017_2034_MOESM1_ESM.pdf]

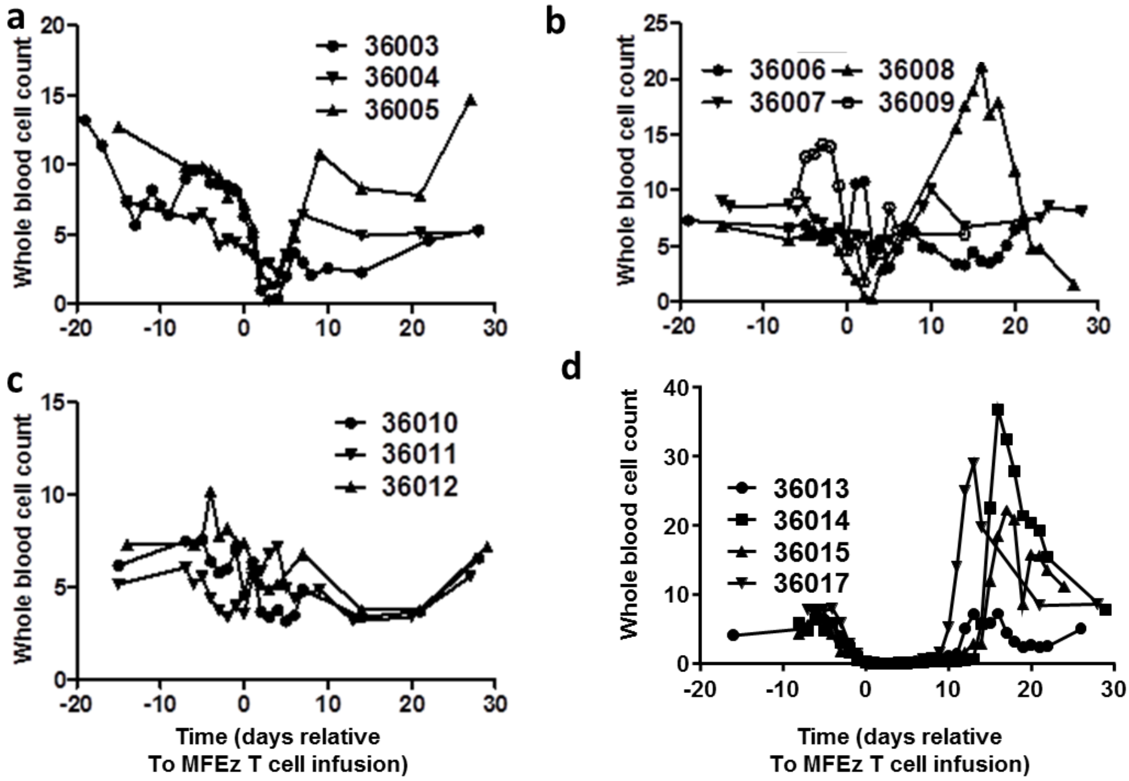

Supplementary Figure 1 An increased intensity of patient pre-conditioning results in prolonged depletion in peripheral blood counts. Whole blood cell counts (x 10^9 cells/L) of patients in (**a**) cohort 1 (**b**) cohort 2 (**c**) cohort 3 and (**d**) cohort 4.

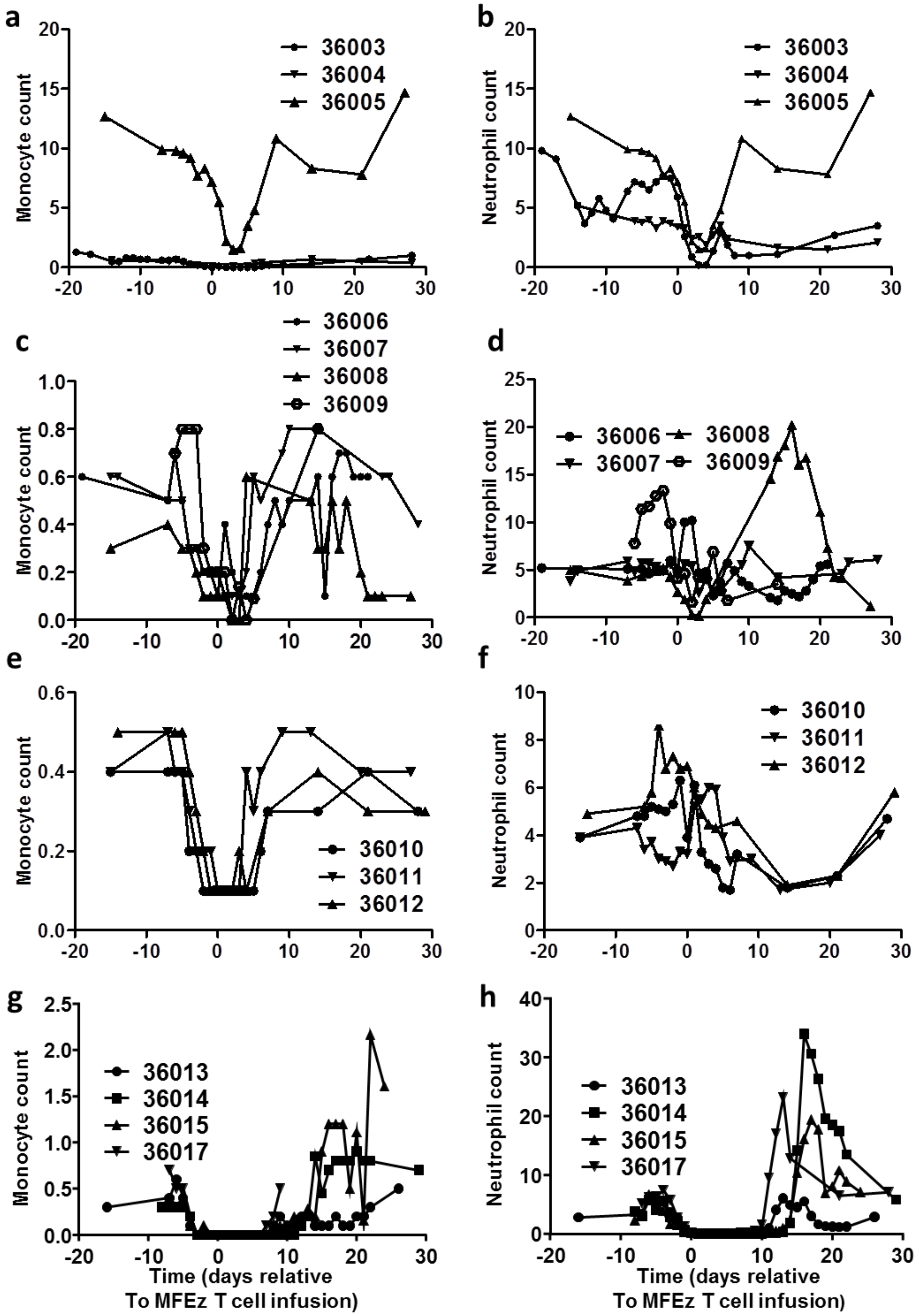

Supplementary Figure 2 Impact of pre-conditioning intensity on monocyte and neutrophil counts. Monocyte counts (x 10^9 cells/L) of patients in (**a**) cohort 1 (**b**) cohort 2 (**c**) cohort 3 and (**d**) cohort 4. Neutrophil counts (x 10^9 cells/L) of patients in (**e**) cohort 1 (**f**) cohort 2 (**g**) cohort 3 and (**h**) cohort 4.

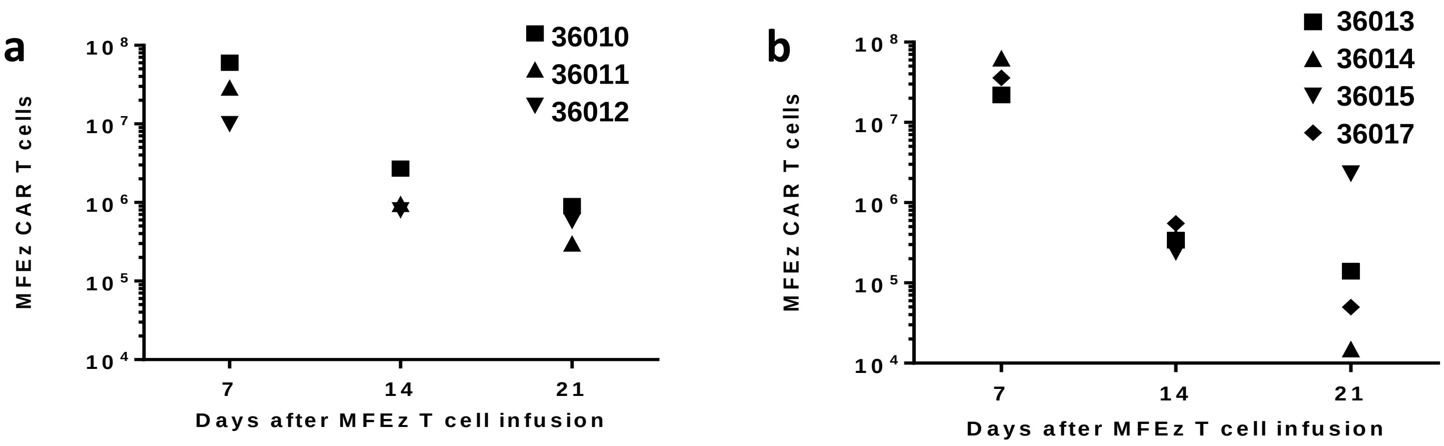

Supplementary Figure 3 An increased intensity of patient pre-conditioning does not impact upon the absolute number of circulating MFEζ CAR T cells. Circulating MFEζ CAR T cell numbers were calculated by using the fraction determined by qPCR data multiplied by the absolute lymphocyte count at the day 7, 14 and 21 time points for patients in (**a**) cohort 3 and (**b**) cohort 4. Numbers were determined using the lymphocyte counts and the relative frequency of MFEζ CAR T cells in Figure 1 to generate an approximation of the number of circulating CAR T cells in each patient.

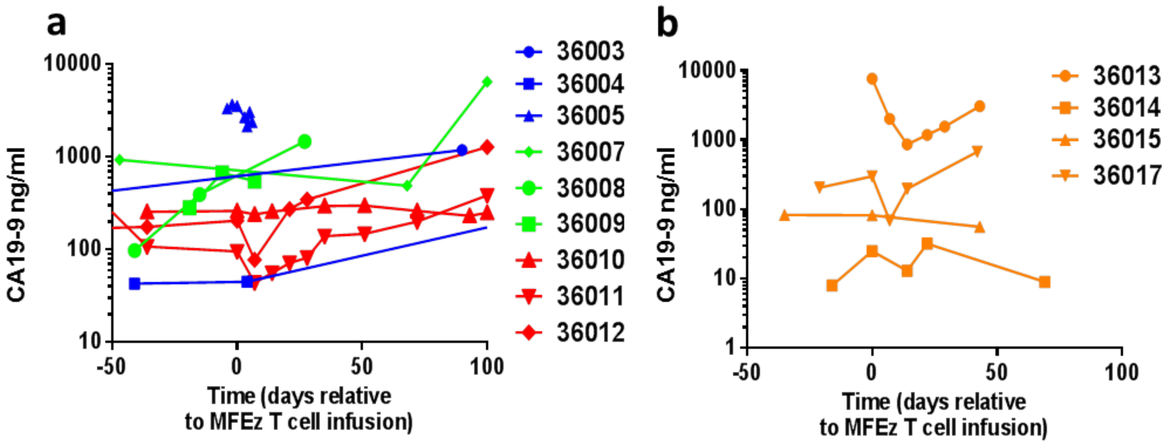

Supplementary Figure 4 CA19-9 serum levels in MFEζ CAR T cell treated patients. Patients in each cohort are color-coded to identify cohorts: cohort 1 in blue, cohort 2 in green, cohort 3 in red and cohort 4 in orange. Serum CA19-9 levels were determined in patient blood samples prior and post MFEζ CAR T cell infusion for (**a**) cohorts 1-3 and (**b**) cohort 4.

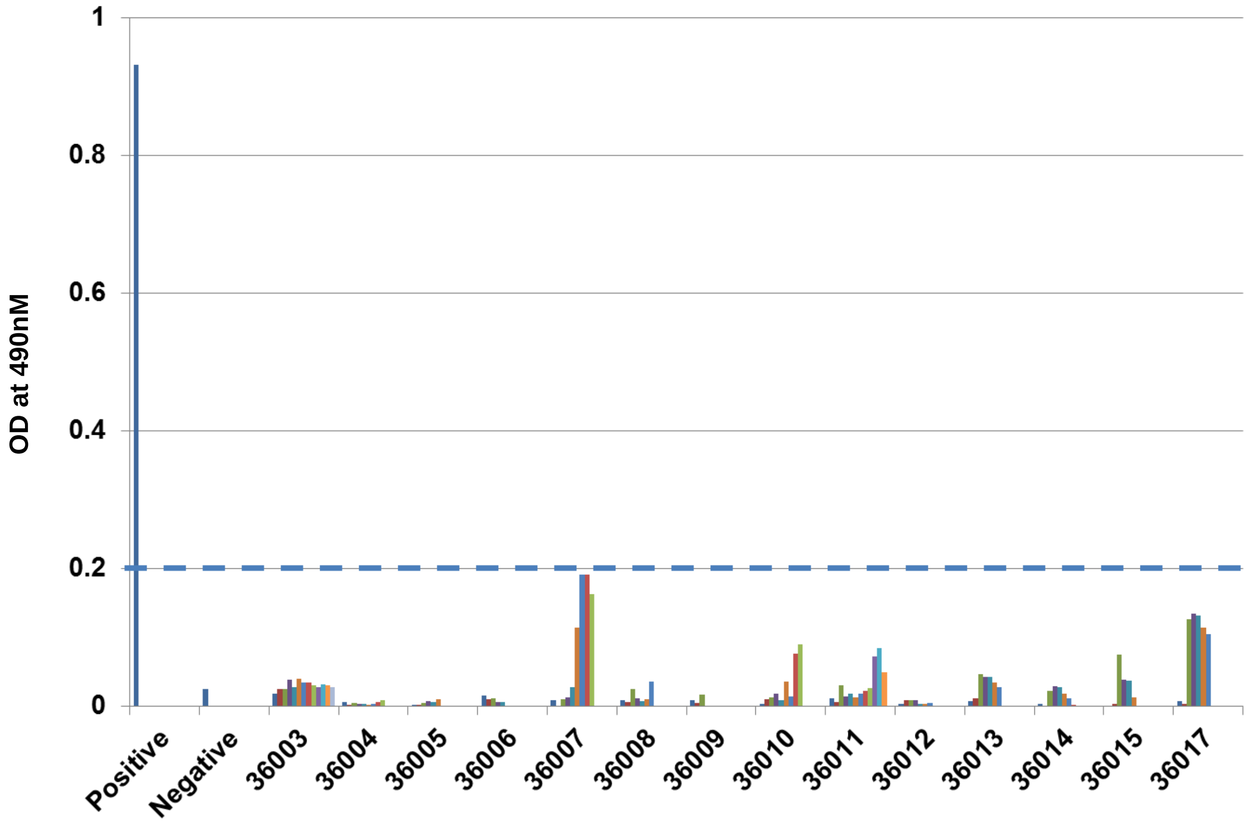

Supplementary Figure 5 Absence of HAMA response was seen in any patient above that of the pre-established lower limit of detection (0.2OD at 490nM). Positive and negative control readings are demonstrated. The different colored bars represent different patient samples taken pre and post CAR T cell infusion.

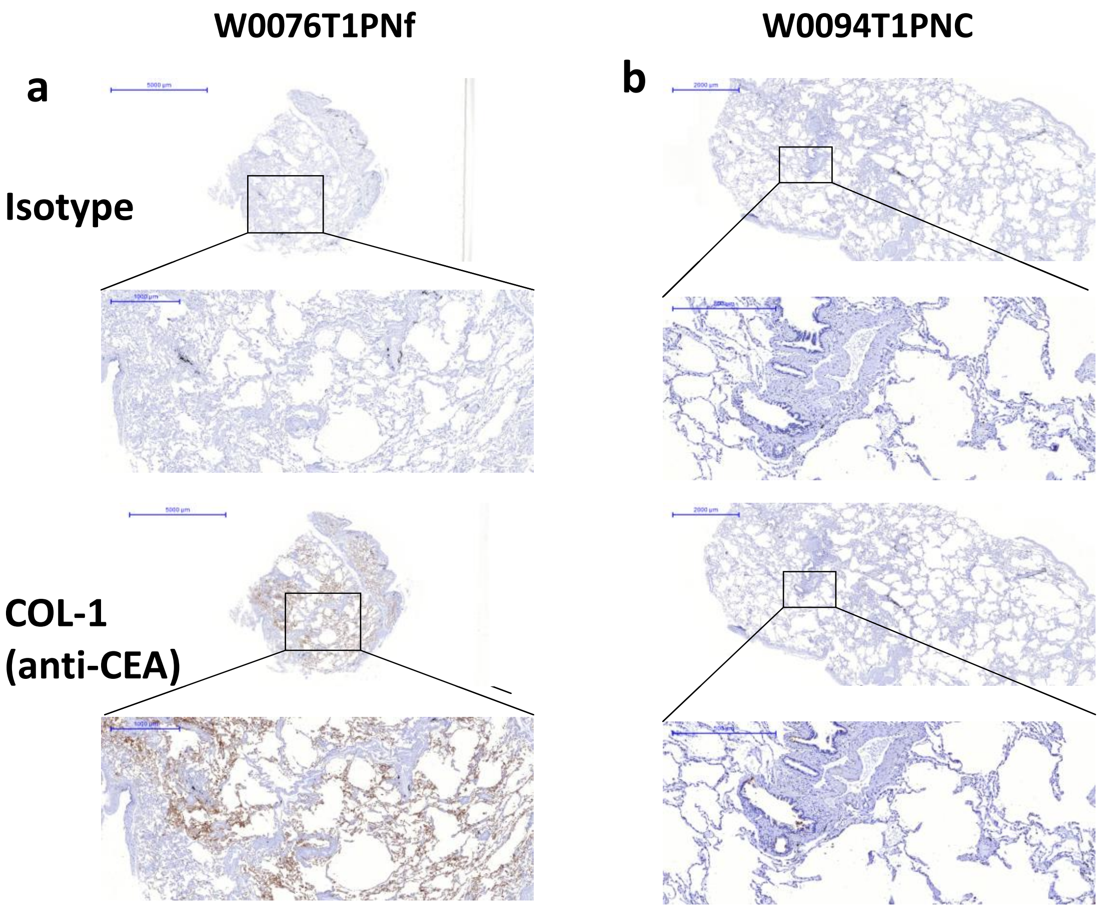

c

Sample	IHC	qPCR
WT00112T1PNf	0/+	+/-
W0665T1PNg	0	+/-
W0081T1PNc	+	+/-
W0076T1PNf	++	+
W00115T1PNd	+	+
W00851PNc	+	+
W0094T1PNc	0/+	-
W0096T1PNc	++/+++	+
W0098T1PNf	+	+

Supplementary Figure 6 CEACAM5 expression on non-cancerous lung tissue samples. Strong CEACAM5^{+} expression by IHC isotype negative control and positive COL1 staining (**a**). Scattered staining (**b**). Summary of level of IHC COL1 staining and qPCR signal (**c**); 0 = No obvious staining of any cells; + = small number of positive cells either localised or scattered through the sample; ++ = Frequent cell staining; +++ = Majority of cells stained.

Cohort	Patient	Adverse events identified at time points prior to MFEζ T cell transfer on day 0. (Max CTCAE Grade) - duration		Adverse events identified at time points after MFEζ T cell transfer on day 0. (Max CTCAE Grade) – duration	
1	36003	-17	Bone and Hip Pain (3)	0	Pelvic Pain (3) – 20 days
				2	Neutropaenia (3) – 1 day
				233	General Deterioration (3)
	36004			1	Hypocalcaemia (3) – 13 days
				2	Leukopenia (4) – 4 days
				3	Hypophosphataemia (3) – 4 days
				4	Lymphopenia (4) – 2 days
				37	Left side pain (3) – 33 days
				55	Abdominal pain (3)
	36005			2	Anaemia (3) – 2 days
2	36006	-7	Fatigue (3)	2	Abdominal pain (3) – 18 days
		-5	Anorexia (3) – 27 days	4	Thrombocytopenia (3) – 3 days
				39	Vomiting (3) – 5 days
				65	Abdominal pain (3)
				65	Vomiting (3)
	36007	-15	Anaemia (3) – 1 day	1	Hypocalcaemia (3) – 13 days
				62	Anaemia (3) – 1 day
	36008			0	Hypotension (3) – 3 days
				1	Neutropaenia (4) – 23 days
				2	Leukopenia (4) – 2 days
				3	Intermittent Hyperbilirubinaemia (3) – 11 days
				3	Hypoalbuminaemia (3) – 25 days
				19	Epistaxis (3) – 2 days
	36009			0	Hyperbilirubinaemia (3) – 13 days
				3	Hypocalcaemia (3) – 10 days
				3	Hyponatraemia (3) – 10 days
				31	Hypoalbuminaemia (3)
3	36010			0	Intermittent pyrexia (3) – 3 days
	36011	-3	Lymphopenia (4) – 12 days	2	Thrombocytopenia (3) – 7 days
				2	Hyperbilirubinaemia (3) – 11 days
				104	Haematemesis (3) – 52 days
				146	Jaundice (3)
				149	Hyperbilirubinaemia (3)
	36012			5	Hypophosphataemia (4) – 5 days
				24	Vomiting (3)
				31	Fatigue (3)
				36	Anaemia (3) – 9 days

Supplementary Table 1 Summary of Adverse Events reported in the MFEζ trial including maximum CTCAE grade and duration of events for cohorts 1-3.

Cohort	Patient	Adverse events identified at time points prior to MFEζ T cell transfer on day 0. (Max CTCAE Grade) - duration		Adverse events identified at time points after MFEζ T cell transfer on day 0. (Max CTCAE Grade) – duration	
4	36013	-6	Nausea (3) – 8 days	1	Neutropenic sepsis (3) – 20 days
		-6	Vomiting (3) – 8 days	1	Intermittent anaemia (3) – 18 days
		-2	Fatigue (3)	1	Thrombocytopenia (4) – 25 days
		-1	Neutropenia (4) – 13 days	2	Hyperbilirubinaemia (3) – 7 days
				3	Intermittent increased respiratory rate (3) – 15 days
				5	Diarrhoea (3) – 20 days
				5	Colitis (*C. difficile* positive) (3)
				5	Pulmonary odema (3) – 12 days
				7	Intermittent bilateral lung shadowing (3) – 12 days
				26	Epigastric pain (3)
				33	Left lower back pain (3)
				94	Death due to disease progression (5)
	36014	-7	Intermittent anaemia (3) – 28 days	0	Intermittent increased respiratory rate (3) – 15 days
		-1	Neutropenia (4) – 15 days	1	Intermittent thrombocytopenia (4) – 12 days
				1	Hypotension (3) – 15 days
				1	Intermittent pyrexia (3) – 15 days
				1	Intermittent hypoxia (3) – 13 days
				2	Dyspnoea (3) – 17 days
	36015	-7	Intermittent nausea (3) – 4 days	0	Intermittent pyrexia (3) – 18 days
		-4	Fatigue (3)	0	Intermittent hypoxia (3) – 18 days
		-1	Neutropenia (4) – 15 days	1	Hypocalcaemia (3) – 40 days
				1	Intermittent hypotension (3) – 12 days
				1	Anaemia (3) – 2 days
				2	Hypophosphataemia (3) – 16 days
				2	Thrombocytopenia (4) – 12 days
				2	Dyspnoea (3) – 17 days
				6	Anorexia (3)
				6	Anaemia (3) – 2 days
				66	Abdominal pain (3) – 31 days
	36017	-1	Neutropenia (4) – 11 days	0	Intermittent pyrexia (3) – 9 days
				0	Intermittent increased respiratory rate (3) – 12 days
				1	Intermittent hypoxia (3) – 9 days
				2	Intermittent thrombocytopenia (4) – 9 days
				3	Intermittent hyperbilirubinaemia (3)
				5	Hypophosphataemia (3) - 5 days

Supplementary Table 2 Summary of Adverse Events reported in the MFEζ trial including maximum CTCAE grade and duration of events for cohort 4.
